# The Sulfated Laminarin Triggers a Stress Transcriptome before Priming the SA- and ROS-Dependent Defenses during Grapevine's Induced Resistance against *Plasmopara viticola*

**DOI:** 10.1371/journal.pone.0088145

**Published:** 2014-02-06

**Authors:** Adrien Gauthier, Sophie Trouvelot, Jani Kelloniemi, Patrick Frettinger, David Wendehenne, Xavier Daire, Jean-Marie Joubert, Alberto Ferrarini, Massimo Delledonne, Victor Flors, Benoit Poinssot

**Affiliations:** 1 UMR 1347 Agroécologie, Université de Bourgogne, Dijon, France; 2 UMR 1347 Agroécologie, INRA, Dijon, France; 3 Laboratoires Goëmar, Saint Malo, France; 4 Dipartimento di Biotecnologie, Università degli Studi di Verona, Verona, Italy; 5 Plant Physiology Section, University of Jaume I, Castellón, Spain; Agriculture and Agri-Food Canada, Canada

## Abstract

Grapevine (*Vitis vinifera*) is susceptible to many pathogens which cause significant losses to viticulture worldwide. Chemical control is available, but agro-ecological concerns have raised interest in alternative methods, especially in triggering plant immunity by elicitor treatments. The β-glucan laminarin (Lam) and its sulfated derivative (PS3) have been previously demonstrated to induce resistance in grapevine against downy mildew (*Plasmopara viticola*). However, if Lam elicits classical grapevine defenses such as oxidative burst, pathogenesis-related (PR)-proteins and phytoalexin production, PS3 triggered grapevine resistance *via* a poorly understood priming phenomenon. The aim of this study was to identify the molecular mechanisms of the PS3-induced resistance. For this purpose we studied i) the signaling events and transcriptome reprogramming triggered by PS3 treatment on uninfected grapevine, ii) grapevine immune responses primed by PS3 during *P. viticola* infection. Our results showed that i) PS3 was unable to elicit reactive oxygen species (ROS) production, cytosolic Ca^2+^ concentration variations, mitogen-activated protein kinase (MAPK) activation but triggered a long lasting plasma membrane depolarization in grapevine cells, ii) PS3 and Lam shared a common stress-responsive transcriptome profile that partly overlapped the salicylate- (SA) and jasmonate-(JA)-dependent ones. After *P. viticola* inoculation, PS3 specifically primed the SA- and ROS-dependent defense pathways leading to grapevine induced resistance against this biotroph. Interestingly pharmacological approaches suggested that the plasma membrane depolarization and the downstream ROS production are key events of the PS3-induced resistance.

## Introduction

Grapevine (*Vitis vinifera*), a major fruit crop worldwide, is affected by many diseases, such as downy mildew (*Plasmopara viticola*), powdery mildew (*Erysiphe necator*) and grey mould (*Botrytis cinerea*). Against these severe diseases, modern sustainable viticulture aims to limit chemical treatments by using alternative strategies. One of them is to trigger grapevine resistance by eliciting its innate immunity.

Plants use two distinct layers of immunity to counterattack microbial infections [1]. The first, PAMP-triggered immunity (PTI), is based on the detection *via* pattern recognition receptors (PRRs) of evolutionarily conserved elicitors, also called pathogen-, microbe- or damage-associated molecular patterns (PAMPs; MAMPs; DAMPs) [2]. However, successful pathogens can secrete effectors that suppress or interfere with PTI, resulting in effector-triggered susceptibility (ETS) [2]. The second level of perception involves the direct or indirect recognition of pathogen effectors by intracellular immune receptors leading to effector-triggered immunity (ETI) [2]. ETI is highly specific and usually accompanied by a hypersensitive response (HR) manifesting as localized cell death at the point of infection [2].

Both PTI and ETI lead to the activation of plant signaling events within minutes to a few hours after perception [3]. Thus, anion effluxes and cytosolic calcium variations are amongst the earliest responses observed in plant cells following elicitor recognition [4], [5]. These ion fluxes contribute to plasma membrane depolarization that can act upstream of cell death. Indeed, anionic channel inhibitors have been shown to block anion efflux and HR triggered by the elicitor cryptogein in tobacco [6], [7]. The ROS, mainly produced by plasma membrane NADPH oxidases [8], [9], together with the activation of MAPK cascade are complementary signaling events leading to a whole transcriptome reprogramming [10]–[12]. Plant hormones such as salicylate (SA), jasmonate (JA), ethylene (ET), and abscissic acid take part in fine-tuning the defense responses [13], [14]. In Arabidopsis, the consensus is that the SA-dependent signaling pathway is required for defense against biotrophs, while the JA/ET pathways are important against necrotrophs [15]. One outcome of these defense signaling pathways is the production of antimicrobial secondary metabolites such as phytoalexins [16] and PR proteins such as β-1,3 glucanases and chitinases [17].

In grapevine, some molecules elicit several of the aforementioned signaling events, *e.g.*, DAMPs like oligogalacturonides (OGs) and PAMPs like the β-1,3 glucan laminarin (Lam) or the *B. cinerea* endopolygalacturonase BcPG1 [18]–[20], while some others like β-aminobutyric acid (BABA) do not [21]. Thus BABA-induced resistance is more mediated by the priming phenomenon [21], [22].

The priming is achieved either *via* exposure to a low amount of pathogen or symbiotic rhizobacteria, or with treatment with molecules such as BABA [22]–[24]. Contrary to elicitation, priming did not trigger notable defense responses in the plant, but upon subsequent challenge by biotic or abiotic stress the cells react with faster and stronger defense responses [25]–[27].

Laminarin (Lam), a β-1,3 glucan polymer (degree of polymerization: DP 33) from the algae *Laminaria digitata,* is able to elicit defense-related events in tobacco and grapevine [18], [28]. Lam treatment also results in partial resistance against *Tobacco mosaic virus* (TMV) or *B. cinerea* and *P. viticola* in tobacco or grapevine, respectively [18], [29]. The chemically sulfated form of Lam, PS3, clearly enhances the tobacco plant immunization against TMV [29]. Similarly, PS3 treatment of susceptible grapevine strongly limits colonization and sporulation of the oomycete *P. viticola* [30]. However, the mode of action differs depending on the plant species. Indeed, in tobacco and Arabidopsis, PS3 triggered plant immunity by direct elicitation of plant defenses including ROS production, the expression of SA- and ET-dependent PR-proteins and phytoalexin synthesis [29]. Conversely, in grapevine, the PS3-induced resistance (PS3-IR) is due to priming of plant defense genes, callose and phenol depositions and HR-like cell death, triggered only after pathogen inoculation [30].

The aim of this study was to decipher the mode of action of PS3 in grapevine. For this purpose, the early signaling events triggered by PS3 were first investigated in grapevine cell suspension. Compared to the well-known elicitor Lam, PS3 did not elicit classical early signaling events but triggered an enhanced and prolonged plasma membrane depolarization. Grapevine transcriptomics analyses were performed on uninfected plants to identify genes that might directly contribute to the mode of action of PS3. Thus, the aims were (i) to determine specific PS3-modulated genes compared to those regulated upon Lam treatment, and (ii) to identify, for the first time in grapevine, gene expression profiles upon SA and JA treatments since these hormones have been shown to play key roles in plant immunity. Thus, a microarray analysis performed at 12 h post-treatment (hpt) led to the identification of 33 genes specifically modulated by PS3 while most of the others were common to Lam and PS3 treatments. In parallel, microarrays studies of SA- or JA-induced transcriptomic changes at 12 hpt allowed the identification of grapevine SA- and JA-marker genes and showed that PS3-transcriptome only partly overlaps those of SA and JA. The PS3-primed grapevine defenses were also investigated after inoculation with the causal agent of downy mildew, *P. viticola.* Our results indicated that PS3 primed the biosynthesis of SA and the expression of SA-marker genes in plants challenged with *P. viticola*. Finally, ROS and anion channels were shown to be key components of PS3-IR.

## Materials and Methods

### Chemical Molecules

The β-glucans Lam and PS3 were prepared in ultra-pure water for cell suspension experiments, or in water with an appropriate adjuvant (0.05%) for experiments realized on plants [30]. Lam, PS3 and the adjuvant were provided by Goëmar and are available on request (http://www.goemar.com). Equal volume of ultra-pure water or 0.05% adjuvant was used as control in cell suspension or plant experiments, respectively. For plant treatments, solutions were sprayed to upper and lower leaf faces until the run-off point. Glucan treatment was applied 1 and 2 days before inoculation with *P. viticola* for experiments realized on leaf discs or on plants, respectively. Adjuvant or β-glucans have no direct toxic effect on *P. viticola* sporangia and zoospores [30].

All pharmacological compounds were purchased from Sigma-Aldrich and dissolved in dimethylsulfoxide (DMSO). Control treatment consists in equivalent volumes of water or DMSO. When used, final DMSO concentration did not exceed 0.25% (v/v). The NADPH oxidase inhibitor diphenylene iodonium (DPI; [21]) and the glibenclamide anion channel blocker (Gli; [6], [7]) were added 30 min before PS3 treatment. All chemicals used were tested for their non-toxicity 4 h and 24 h after treatment on grapevine cells and herbaceous cuttings, respectively.

### Cell Culture Treatments

Grapevine (*V. vinifera* cv. Gamay) cell suspensions were maintained as described by Vandelle *et al.* [31]. Cells were collected during the exponential growth phase, were washed with the suspension buffer (175 mM mannitol, 0.5 mM K_2_SO_4_, 0.5 mM CaCl_2_, and 10 mM MES, pH 5.3), and then was resuspended at 0.1 g ml^−1^ fresh weight of cells (FWC). After 1 h of equilibration (150 rpm, 24°C), grapevine cells were treated with β-glucans and analyses of ROS production, cytosolic Ca^2+^ concentration variations, or MAPK phosphorylation were performed.

### Free Cytosolic Calcium Concentration Variation Analysis

Measurements of the [Ca^2+^]_cyt_ were based on aequorin bioluminescence using a luminometer (Lumat LB 9507, Berthold, Evry, France). *In vivo* reconstitution of aequorin was performed by the addition of 6 µl of coelenterazine (5 mM stock solution in DMSO) to 10 ml of aequorin-transformed cell suspension for at least 3 h in the dark. Luminescence was recorded continuously and data converted to free cytosolic calcium concentration using the calibration equation described in Vandelle *et al*. [31].

### H_2_O_2_ Production Measurement

H_2_O_2_ production was assessed using a luminol chemiluminescence assay with a luminometer (Lumat LB 9507, Berthold, Evry, France). Aliquots (250 µl) of cell suspension were analyzed after the addition of 300 µl of H50 medium (50 mM Hepes, 175 mM mannitol, 5 mM CaCl_2_, 0.5 mM K_2_SO_4_; pH 8.5) and 50 µl of 0.3 mM luminol. Luminescence emission was monitored and recorded every 10 s. Relative luminescence units were converted in nmol of H_2_O_2_ per gram of fresh weight cell (FWC), after the establishment of a H_2_O_2_ reference range with untreated cell suspension.

### Plasma Membrane Depolarization Measurement

Cells were equilibrated in the dark for 2 h in suspension buffer supplemented with 10 µM DiBAC_4_ (Bis(1,3-dibarbituric acid)-trimethine), as described in Dubreuil-Maurizi *et al*. [32]. Five hundred microliters were transferred per well into a 24-well plate and DiBAC_4_ fluorescence was recorded continuously at 10s intervals, using a fluorimeter (Fluoroskan Ascent Fluorometer, Labsystems, Helsinki, Finland) with λ_ex_ = 485 nm and λ_em_ = 525 nm. Fluorescence was expressed as relative fluorescence units (RFU).

### Western Blot Analyses

Fifteen µg of protein per sample were solubilized in Laemmli buffer, submitted to 12% SDS-PAGE before Western blotting. Nitrocellulose membrane is pre-incubated first during 2 h at room temperature with TBST buffer (10 mM Tris-HCl, 150 mM NaCl, 0.05% Tween-20, pH 7.5) and 1% BSA; then incubated with primary antibody (Cell Signalling): anti non phosphorylated human ERK1/2 (total MAPK, inactive) or anti-phospho Thr202/Tyr204 peptide of human ERK1/2 (phosphorylated MAPK, active) mouse antibody, 1/3000 diluted in TBST buffer during 1h30 under shaking. After 3 washes with TBST buffer for 10 min each time, membrane is incubated with goat HRP-conjugated anti-mouse secondary antibody (Sigma-Aldrich), 1/6000 diluted in TBST buffer. After 3 washes with TBST buffer for 10 min each time, MAPK were detected using ECL detection kit (Pharmacia Biosciences).

### Plant Materials

The *V. vinifera* (cv. Marselan) plants, susceptible to *P. viticola*, were obtained from herbaceous cuttings placed in individual pots containing a mixture of peat and perlite (4/1, v/v), and grown in a glasshouse at a temperature of 24/18°C (day/night) with a photoperiod of 16 h light. Plants were watered daily and fertilized once a week. Leaf material was collected from the 2^nd^ and 3^rd^ youngest fully expanded leaves where the level of PS3-induced resistance has been demonstrated to be the highest [33].

### Pathogen Inoculation and Disease Assessment

Two days after treatment, leaves of *V. vinifera* plants (cv. Marselan) were inoculated with *P. viticola* by spraying onto the lower face a freshly prepared sporangia suspension at 10^4^ sporangia ml^−1^ [33]. The mycelium development was analyzed by aniline blue staining. Foliar discs from the youngest 2^nd^ and 3^rd^ fully expanded leaves were fixed overnight at room temperature in pure methanol solution before clarification in chloral hydrate solution (2.5 g l^−1^). Samples were rinsed with phosphate buffer (0.1 M Na_2_HPO_4_, pH 8.0), then stained overnight with aniline blue staining solution (0.05% prepared in phosphate buffer) and observed by epifluorescence microscopy under UV (λ_ex_–λ_em_: 340–380 nm).

Concerning leaf disks assays, leaf disks (*V. vinifera* cv. Marselan) were floated (lower face in contact with solution) on the chemical solutions (200 µM Gli, 10 µM DPI or diluted DMSO) during 24 h, washed 3 times with water before the second treatment (adjuvant, or 2.5 g l^−1^ PS3) during 24 h. The leaf disks were rinsed 3 times in water and then transferred (lower face uppermost) onto moist Whatman filter paper in a Plexiglas box before inoculation with *P. viticola* (20 µl at 10^4^ sporangia ml^−1^ per disk).

Disease intensity was assessed by measuring the leaf area covered by *P. viticola* sporulation at 8 dpi, as described by Trouvelot *et al.* [30].

### Localization of H_2_O_2_ and Callose Deposition in Treated Leaves

*In situ* H_2_O_2_ production was revealed by brown precipitates after 3,3′-diaminobenzidine (DAB) staining. Gli, DPI, or DMSO (control) was administered by petiolar absorption during 24 h, and then 2^nd^ and 3^rd^ fully expanded leaves were transferred into water. Leaves were sprayed with adjuvant or PS3 (5 g l^−1^) until the run-off point and inoculated 1 day later with *P. viticola* (10^4^ sporangia ml^−1^) to upper and lower leaf faces. Leaves were harvested at different time points and transferred for an additional 5 h period (80 µmol m^−2^ s^−1^, 50% relative humidity ±10, 22°C) to DAB solution (1 g l^−1^). Harvested leaf discs were bleached first with pure methanol, then chloral hydrate solution (2.5 g l^−1^). H_2_O_2_ is visualized as a reddish-brown deposit in DAB-treated leaves. Callose deposition was revealed by aniline blue staining as previously described [21]. Briefly, clarified leaf disks were stained in 0.05% aniline blue (in phosphate buffer) overnight and then were mounted on microscope slides in the same solution. Pathogen structure and callose deposition were observed in blue by epifluorescence microscopy under UV (λex = 340 nm, λem = 380 nm, stop filter LP 430 nm, Leica).

### Microarray Analysis

Triplicate samples were collected on grapevine plants spray treated with 5 g l^−1^ PS3, 5 g l^−1 ^Lam, the mock 0.05% Adj, 1 mM SA, 40 µM JA or the corresponding control (0.1% DMSO) at 12 hpt. For each time point, the 2^nd^ and 3^rd^ youngest full-sized leaves of 4 independent plants were harvested. Then, RNA samples from three independent biological experiments (n = 3) were extracted according to Reid *et al.* [34], purified by LiCl precipitation and analyzed using an RNA 6000 nano kit (Agilent Technologies, Waldbronn, Germany). One µg of RNA was processed using AminoAllyl MessageAmpTM aRNA (Ambion, Life technologies, Saint Aubin, France) and hybridized to a Combimatrix Grape Array 1.2 (24562 unigenes) according to the manufacturer’s protocols. Microarrays were scanned using ScanArray 4000 XL (PerkinElmer Life Sciences, Waltham, MA, USA). Data extracted with Microarray Imager (Combimatrix, Irvine, CA, USA) were normalized by median scaling before deposition in NCBI GEO database (ID: GSE42972). Differentially-expressed genes were identified by pairwise comparison of plants treated (PS3, Lam or SA, JA) *versus* control plants (Adj or DMSO ) as described by Tsai *et al.* [35]. GO enrichment analysis was performed on PS3-up-regulated genes with the AgriGO software [36].

### Quantitative Reverse-transcription PCR (qPCR)

For qPCR analysis, 4 independent biological experiments were performed as described above (n = 4). Briefly, plants were first sprayed either with adjuvant only or with adjuvant plus PS3 (5 g l^−1^) and subsequently inoculated with the pathogen. For each experiment, the 2^nd^ and 3^rd^ youngest fully expanded leaves from 4 individual plants were sampled and pooled per treatment at 0, 1, and 2 days post inoculation (dpi). Then, total RNA was extracted as described above. cDNA was synthesized using Superscript III Reverse Transcriptase kit (Invitrogen, Life technologies, Saint Aubin, France), random hexamers, and 2 µg of DNA-free total RNA according to the manufactureŕs instructions. The qPCR experiments were carried out with the ABsolute™ QPCR SYBRGreen ROX Mix (Thermo Scientific, Waltham, MA, USA), with a final primer concentration of 500 nM, in a LightCycler480 (Roche, Meylan, France) using a thermal cycling profile of 95°C 15 min; 40 cycles of 95°C for 20 s, 60°C for 30 s, 72°C for 30 s. The melting/dissociation curve of each reaction was checked to ensure a single amplicon was produced. The mean cycle threshold (Ct) value of a sample's technical triplicates was used for the further analysis. The relative gene expression was determined with the comparative Ct method [37]: 2^–ΔΔCt^; where ΔΔCt = ΔCt (treated sample) – ΔCt (control sample) and ΔCt = Ct (target gene) – Ct (reference gene). See Table S4 for the sequences of the used primers, including those of *VATP16* as the reference gene [38].

### Quantification of JA and SA Metabolites by LC-MS

Fifty mg of freeze dried leaves were harvested in parallel of microarray samples and homogenized after addition of a mixture of internal standards containing 100 ng dihydro-JA and D_6_-SA [39]. After centrifugation the supernatant was purified by organic partitioning, as previously described [40], [41]. A dried aliquot was re-suspendended in 1 ml of MeOH: H_2_O (10∶90) and injected into the HPLC system. Analyses were carried out using a Waters Alliance 2690 HPLC system (Milford, MA, USA) with nucleosil ODS reversed-phase column (100×2 mm i.d.; 5 lm; Scharlab, Barcelona, Spain) coupled to a Quatro LC (quadrupole-hexapole-quadrupole) mass spectrometer (Micromass). Data was quantified after comparative analysis with standards using Mass Lynx (v 1.4, Mycromass) software.

### Statistical Analysis

In order to compare *P. viticola* infection levels, analysis of variance (ANOVA) was performed with the statistical program Stat-Graphics 5.1 (Manugistic, Inc., Rockville, MD, USA) using the least significant difference (LSD) test to detect significant differences (P<0.05) between treatments.

For microarray experiments, differentially expressed transcripts were identified by pairwise comparison [35] of three independent experiments (n = 3 per treatment) with a fold change cut-off ≥2 or ≤0.5 at least in one condition, and a FDR of 5% (*P*<0.05; *t* test). *P* values are indicated in each sheet of the Table S1.

For qPCR experiments, transcripts differentially expressed between PS3- or Adj-treated and subsequently infected plants were identified by comparing means of the data obtained in each modality (technical triplicates of 4 biological replicates; n = 12), using unpaired heteroscedastic Student’s *t* test (*P*<0.05).

## Results

### β-glucans Trigger Grapevine Immunity against *Plasmopara viticola*

Compared to water, the treatment of *V. vinifera* (cv. Marselan) with the control adjuvant (Adj) had no significant effect on disease severity (Figure S1). Conversely, β-glucans, Lam or PS3, clearly reduced the sporulation of *P. viticola* (Figure S1A). Interestingly, PS3 induced a stronger resistance by reducing the *P. viticola* sporulating area by 84% compared to 60% with Lam (Figure S1B).

### PS3 does not Elicit Early Signaling Events Except a Strong Plasma Membrane Depolarization

**Figure 1:**
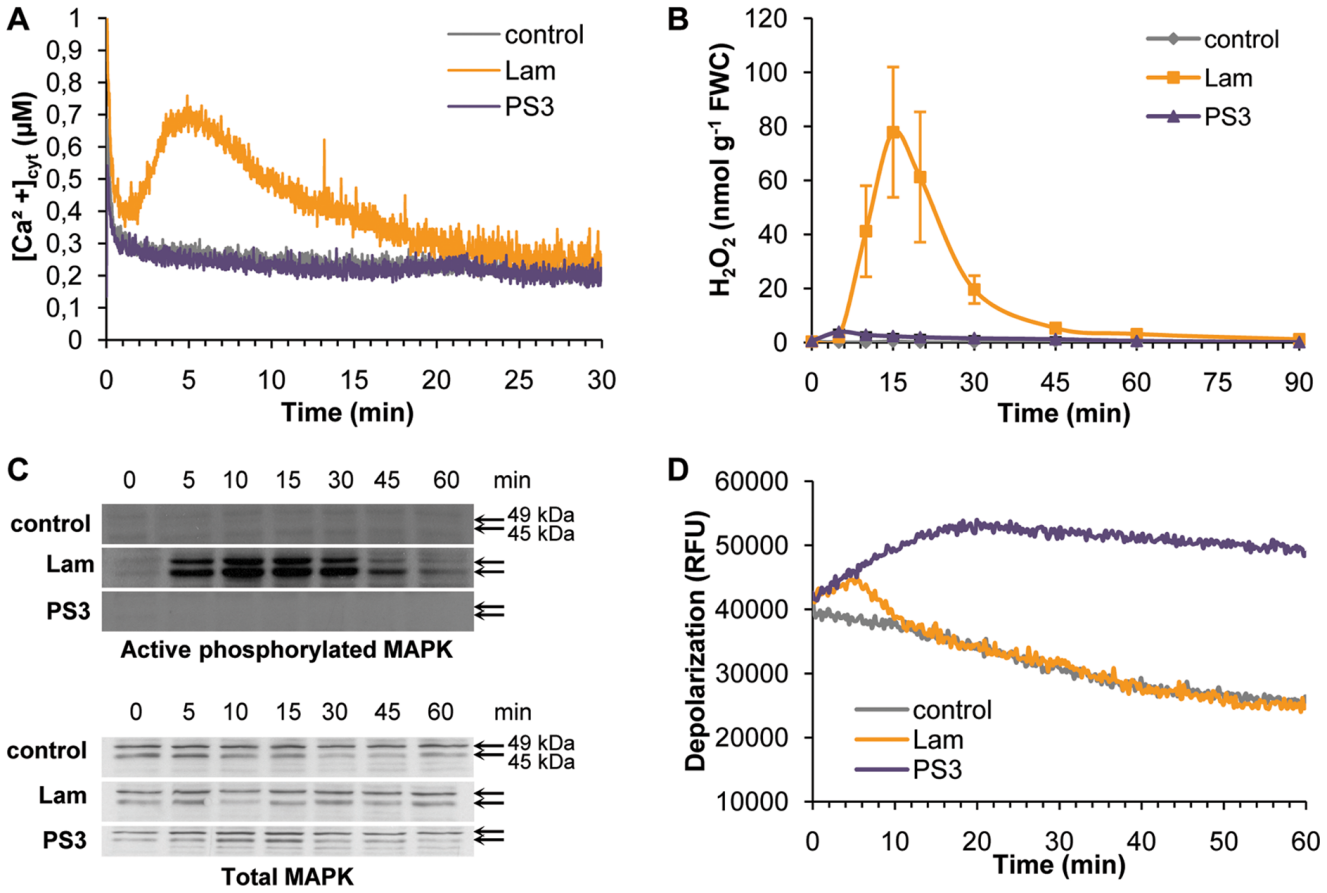
Effects of β-1,3 glucans on early signaling events in grapevine cells. *V. vinifera* cell suspensions were treated with PS3 (2 g l^−1^), Lam (2 g l^−1^) or water (control). **A.** Cytosolic [Ca^2+^] variations in aequorin expressing grapevine cells. **B.** H_2_O_2_ production measured by luminol chemiluminescence assays. FWC: fresh weight of cells. **C.** MAPK activation revealed by western blotting using an antibody raised against active phosphorylated MAPK or total MAPK. **D.** Plasma membrane depolarization revealed by fluorescence of the DIBAC_4_ probe in grapevine cells. RFU: relative fluorescence unit. Results are from one of three representative experiments (n = 3).

Given the role of free Ca^2+^ as a secondary messenger in numerous plant signaling pathways, variations in [Ca^2+^]_cyt_ were investigated using transformed grapevine cells producing apoaequorin addressed to the cytosol. The elicitor Lam induced a rapid and transient increase in [Ca^2+^]_cyt_, whereas in PS3-treated and control cells no increase was detected during the 30 min assay period (Figure 1A). The Lam-induced increase in [Ca^2+^]_cyt_ started after 1 min, peaked at 0.7 µM after 5 min, and returned slowly to the background level (0.25 µM ) within 25 min.

As previously observed [18], Lam induced a quick and transient H_2_O_2_ production. The oxidative burst started 5 min after Lam application and the amount of detected H_2_O_2_ peaked at 80 nmol g^−1^ FWC after 15 min, and proceeded to decline to its initial level of 3 nmol g^−1^ FWC within 45 min (Figure 1B). The very low H_2_O_2_-concentration measured after PS3-treatment was comparable to the one detected in control cell suspension throughout the 90 min assay (Figure 1B).

Total MAPK were first detected with a western blot using an antibody raised against human non-activated ERK1/2 in control, Lam and PS3-treated grapevine cells. It revealed two MAPKs with relative molecular masses of 45 and 49 kDa, respectively, with similar intensities, indicating an equal protein loading (Figure 1C, lower panel). Then, the putative β-glucan-induced phosphorylation of the two detected MAPK in grapevine cells was revealed by western blotting, using an antibody raised against a phosphorylated ERK1/2 peptide. As previously observed [18], phosphorylated MAPK of 45 kDa and 49 kDa were detected between 5 to 45 min post-treatment with Lam, but not in control cells (Figure 1C, upper panel). Interestingly, the phosphorylation of these two MAPKs was undetectable in PS3-treated cells (Figure 1C, upper panel).

The ability of Lam and PS3 to induce ionic movements through plasma membrane was tested with the voltage-sensitive lipophilic anionic fluorophore, DiBAC_4_. Following Lam or PS3 treatment, a rapid increase in fluorescence occurred, indicating plasma membrane depolarization (Figure 1D). With Lam, a transient increase peaked at 6 min then returned to control levels within the next 6 min. With PS3, the fluorescence strongly increased during the first 20 min before reaching a plateau until the end of the assay (Figure 1D). This data indicate that while Lam induces a transient plasma membrane depolarization, PS3 triggers an important and long lasting depolarization of the plasma membrane.

### PS3 and Lam Share a Common Stress-responsive Transcriptome that Partly Overlaps the SA- and JA-dependent Ones

**Figure 2:**
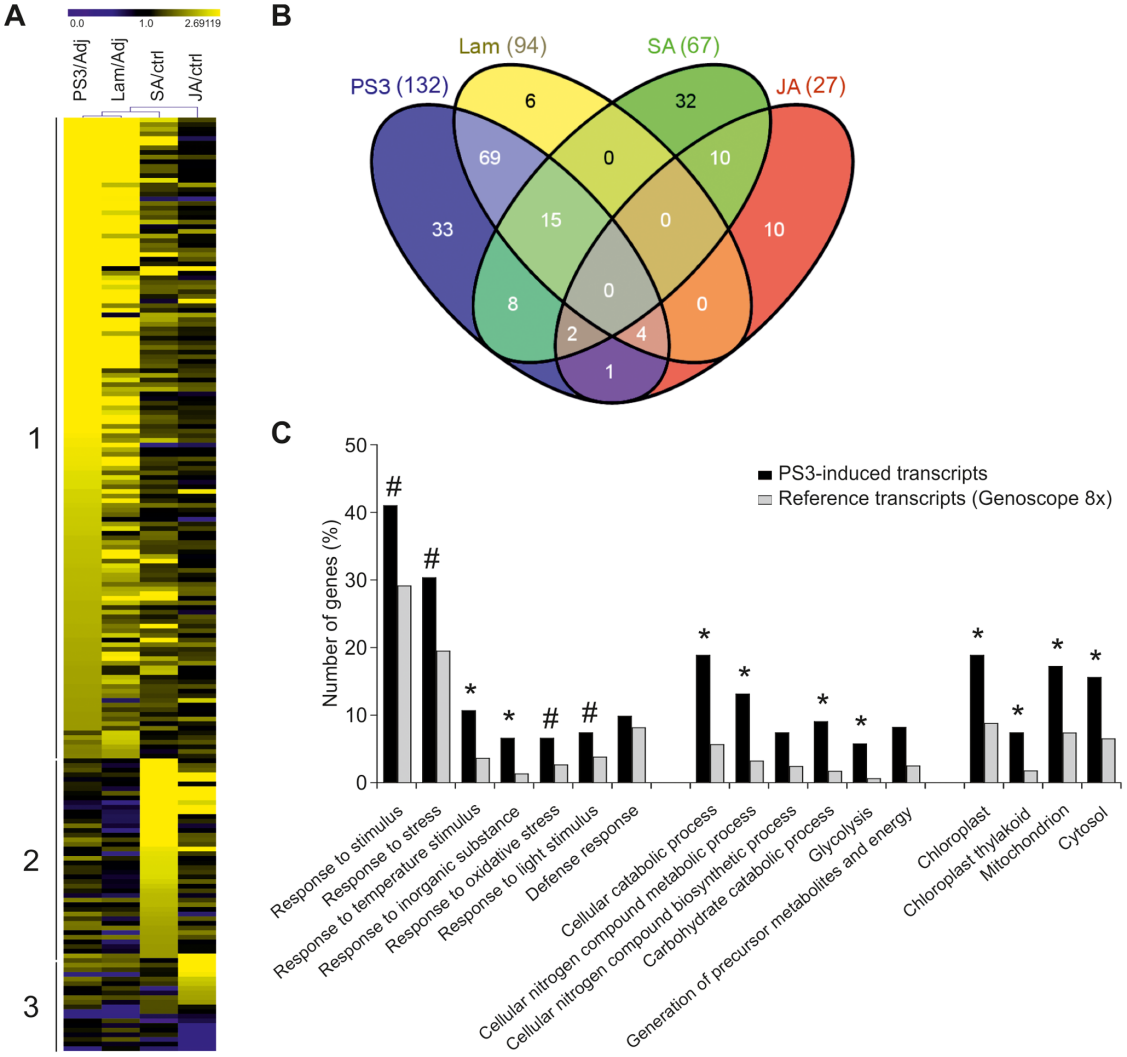
PS3- and Lam-treated plants share a common stress transcriptome that partly overlaps the SA- and JA-dependent ones. **A.** Hierarchical clustering analysis of genes expressed in plants sprayed with PS3 (5 g l^−1^), Lam (5 g l^−1^), salicylic acid (SA, 1 mM), or jasmonic acid (JA, 40 µM), 12 h post-treatment (hpt). Three independent biological experiments were performed (n = 3) and hierarchical clustering was realized with TIGR MeV software [42] after normalization with the corresponding controls: adjuvant (0.05%) for PS3 and Lam and DMSO (0.1%) for SA and JA. **B.** Venn diagram showing specific and common genes regulated by PS3, Lam, SA or JA at 12 hpt (see Table S2 for gene identifiers). **C.** Gene Ontology enrichment analysis of the PS3-up-regulated genes at 12 hpt realized with the AgriGO software [36]. An asterisk (*) implies statistical significance between PS3-induced transcripts *vs* reference transcripts (Fisher's exact test with Benjamini-Yekutieli false detection rate correction, *Q*<0.05; Fold-change cut-off 2 used). A hash mark (#) indicates a significant difference between PS3-induced transcripts *vs* reference transcripts when a more stringent fold-change cut-off of 4 was used.

Combining data from the 18 hybridizations realized for the microarray experiment, 200 genes were significantly modulated by at least one of the treatments (*P*<0.05). The relative fold changes were determined compared to their respective control: 0.05% adjuvant for PS3 and Lam, and 0.1% DMSO for SA and JA. Based on these significantly modulated genes, a hierarchical clustering revealed three different transcriptomic clusters (Figure 2A). The first cluster shows genes co-induced by the 2 β-glucans Lam and PS3 (group 1); the second corresponds to genes modulated after SA treatment (group 2) and the third one is specific to JA treatment (group 3). Among the up-regulated genes (FC>2), 132, 94, 67 and 27 genes were identified after PS3, Lam, SA or JA treatment, respectively (Figure 2B, Table S1). Conversely, 1, 4, 2, and 9 genes were respectively down-regulated after PS3, Lam, SA or JA application (Table S1; FC<0.5).

Interestingly, clustering of genes indicate that PS3 and Lam shared similar expression profiles (Figure 2A). Indeed, 67% (88/132) of genes modulated by PS3 were also up-regulated in response to Lam (Figure 2B; Table S2). Among the commonly modulated genes by the β-glucans, PS3-treated plants generally showed a stronger up-regulation compared to the expression level detected in Lam-treated plants (Table S1). In addition to the 88 common genes induced by Lam and PS3, PS3 also induced 44 other genes: 33 unique to PS3 treatment, 8 in common to SA, 1 in common to JA and 2 commonly induced by PS3, SA and JA (Table S2). Thus compared to Lam, PS3- up-regulated more genes and the induction folds were globally higher. Among the 33 genes specifically induced by PS3 (Figure 2B, Table S2), many encode proteins involved in signaling such as *Phospholipase C*, *Calmodulin*, *CBL-interacting protein kinase (CIPK 14), serine/threonine-protein kinase (AFC2)* and the transcription regulators *NAC78*, *AP2/ERF*, *Jumonji*, *Squamosa binding protein* and *ankyrin repeat 2 (AKR2)*. The *protein phosphatase PP-X 1* and *ubiquitin-conjugating enzyme 32*, known to be signaling repressors in other species, might also play an important role for the PS3-mediated priming phenomenon.

To go further on the PS3 mode of action, a GO enrichment analysis revealed that the PS3-up-regulated transcripts are enriched in organelle-tagged genes, particularly encoding chloroplast and mitochondrion targeted proteins (Figure 2C, Table S3). Concerning the biological processes, the most PS3-up-regulated genes (Figure 2C; FC>4, # symbol) indicated an enrichment in stimulus-responsive genes known to respond to abiotic stimulus (light and temperature) such as the *Rubisco Activase, Heat shock cognate 70 kDa protein 2 (HSC70-2), Heat shock protein 83 (HSP83),* inorganic substance (*Metal-nicotianamine/oligopeptide transporter YSL1*) and oxidative stress (*Glutathione S-transferase GST6*). Using a larger cut-off (FC>2, * symbol), the GO-enrichment analysis (Figure 2C, Table S3) clearly highlighted genes involved in carbohydrate catabolic process, particularly in glycolysis (*Phosphofructokinase, Fructose-biphosphate aldolase, Phosphoglycerate mutase, Glyceraldehyde-3-phosphate dehydrogenase, Pyruvate kinase*) and in nitrogen compound biosynthesis (*nucleotidyltransferase, UMP synthase, cytidine deaminase, glutamine synthetase, aspartate aminotransferase*). However, the “defense response” biological process is not significantly enriched 12 hpt with PS3 (Figure 2C, Table S3).

Moreover, the genes Nucleoredoxin-1 (NRX1), transcription factor WRKY40 (VvWRKY3), β-1,3 glucanase (PR-2), Enhanced Disease Susceptibility 1 (EDS1), Thaumatin-like protein 3 (PR-5), and Pathogenesis-related 1 (PR-1) were found to be specifically induced by SA whereas Jasmonate ZIM-domain protein (JAZ1), Fatty acid hydroperoxide lyase (HPLA), and Allene oxide cyclase (AOC1) could be considered as JA-marker genes in grapevine (Figure 2B, Table S2). At 12 hpt, PS3 is unable to significantly up-regulate all these above-mentioned specific SA- or JA-marker genes (FC>2; P<0.05). However, a few genes were co-regulated by SA or JA and PS3. Indeed, 5% (7/132) were common between PS3 and JA and 19% (25/132) between PS3 and SA (Figure 2B, Table S2). For instance, the putative SA-Methyl Transferase 1 (SAMT1), Carboxylesterase HSR203J like, Receptor-like kinase (RLK1), two Glutathione S-transferase, (GST 23-like and homologue to AtGST6), Avr9 elicitor response protein and Metal-nicotianamine/oligopeptide transporter (YSL1) were co-induced by SA and PS3 suggesting that the SA-dependent pathway might be partly involved in the PS3 mode of action.

To better understand the role of the SA- and JA-dependent pathways, SA and JA metabolites were quantified after PS3 and Lam treatment on uninfected grapevine plants. LC-ESI-MS/MS analysis revealed that PS3 was not able to elicit any significant SA and JA accumulation at 12 hpt and until 48 hpt (Figure S2). However, compared to PS3, Lam seems to elicit a transient increase in JA at 24 and 36 hpt (Figure S2).

### PS3 Primes the SA-dependent Defense Pathway during *P. viticola* Infection

To characterize the putative involvement of phytohormones during PS3-IR, plants were treated with PS3 and 2 days later inoculated with downy mildew (0 dpi at 48 hpt). SA and JA contents were quantified by LC-ESI-MS/MS from 0 to 8 dpi.

**Figure 3:**
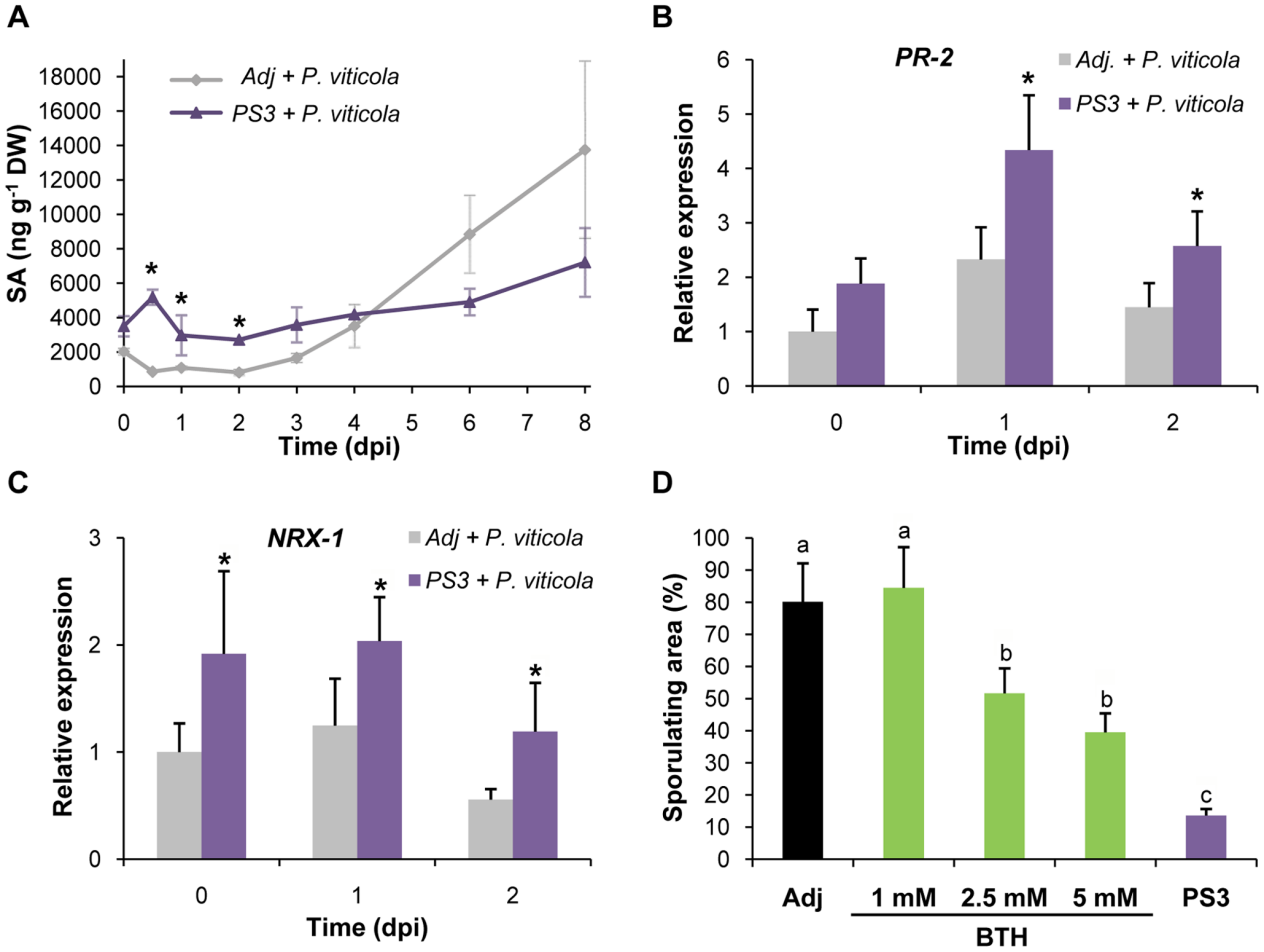
Priming of the grapevine SA-dependent defenses during PS3-IR to *P. viticola*. **A.** Priming of the SA accumulation in PS3-treated plants during *P. viticola* infection. Endogenous total SA quantified by LC-MS in PS3- or adjuvant- (Adj) treated plants after inoculation with *P. viticola* during 8 dpi. Plants were sprayed with PS3 (5 g l^−1^) or adjuvant (0.05%) and, 2 days later, inoculated with *P. viticola* (10^4^ spores ml^−1^). Asterisks indicate significant differences (n = 3, *P*<0.05, student *t* test). DW: dry weight. **B, C.** Primed expression of SA-marker genes encoding a β-1,3 glucanase (*PR-2*) and nucleoredoxin (*NRX-1*) revealed by qPCR in plants treated with PS3 (5 g l^−1^) or Adj (0.05%) at 0, 1 and 2 dpi. Four independent biological experiments were performed and, in each experiment, the 2nd and 3rd youngest leaves of four plants were sampled and pooled. Means of relative expression after PS3 or Adj treatment were compared within a time point. Asterisks indicate significant differences using unpaired heteroscedastic Student's *t*-test (*P*<0.05).The relative expression in Adj at 0 dpi was set as 1 and the others were adjusted accordingly. The error bars represent the standard error of the mean. **D.** The SA analogue benzothiadiazole (BTH) induces grapevine resistance against *P. viticola*. Leaf sporulating area evaluated after 8 dpi. Different letters indicate statistically significant differences (*P*<0.05; ANOVA followed by LSD test). Data are representative of three independent biological experiments (n = 3).

Upon *P. viticola* inoculation, SA concentration detected in control plants (Adj+*P. viticola*) was stable until 3 dpi (<2000 ng g^−1^, Figure 3A), then raised quickly to reach 13800 ng g^−1^ at 8 dpi, when sporulation appeared. Compared to the control plants, a higher SA content, which peaked to 5200 ng g^−1^ at 0.5 dpi, was maintained until 3 dpi in PS3-treated and infected plants (PS3*+ P. viticola*). Then, SA concentration continued to rise slightly but less intensely than in control plants until 8 dpi. At this time point, pathogen spreading and sporulation were undetectable (Figure S1A). In parallel, a non-significant variation in JA content was detected in inoculated PS3-treated compared to adjuvant-treated plants (data not shown).

To gain further insight into the involvement of SA signaling during PS3-IR, expression of 2 SA-marker genes identified in this study (Table S2) was followed by qPCR. As *PR-1* was not highly induced by SA (Table S2), expression of genes encoding the *β-1,3 glucanase* (*PR-2*) and *Nucleoredoxin 1* (*NRX-1*) was followed after 0, 1 and 2 dpi in inoculated PS3- and adjuvant-treated plants. Figures 3B and C indicated that both genes were transiently up-regulated at 1 dpi but that PS3-treated plants showed a significantly higher expression level of *PR-2* and *NRX-1.* These data indicate that, in the early steps of *P. viticola* inoculation, PS3 primed a higher SA production correlated with the expression of 2 SA-marker genes.

To confirm the putative involvement of SA in the PS3-IR against *P. viticola*, grapevine plants were treated with the well-known SA synthetic analogue benzothiadiazole (BTH). Our results indicated that BTH was able to trigger grapevine resistance against downy mildew in a dose dependent manner, even if PS3 was always a more efficient resistance inducer (Figure 3D).

### The Primed ROS-dependent Defense Pathway is Involved in Grapevine Triggered Immunity against *P. viticola* Infection

**Figure 4:**
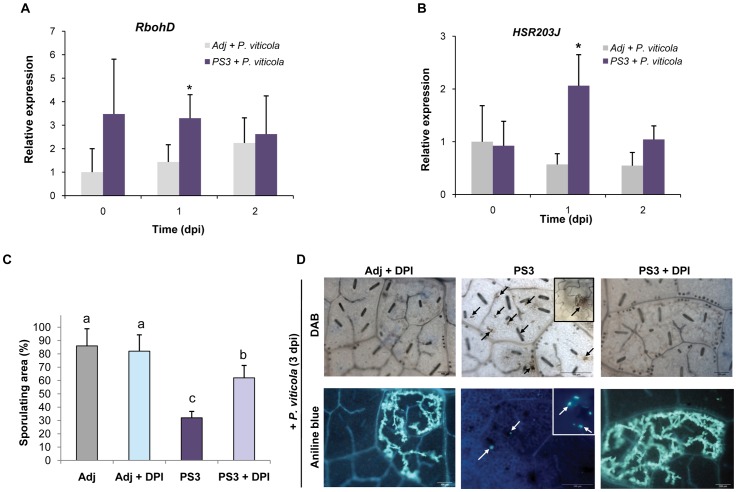
Priming of ROS and HR-like cell death are key defenses during PS3-IR to *P. viticola*. **A, B.** Primed transcript accumulation of genes encoding a NADPH-oxidase (*RbohD*) and a HR-related lipase (*HSR203J*) revealed by qPCR in plants treated with PS3 (5 g l^−1^) or Adj (0.05%) at 0, 1 and 2 dpi. Means of relative expression after the two treatments derived from four independent biological experiments were compared within a timepoint. Asterisks indicate significant differences using unpaired heteroscedastic Student's *t*-test (*P*<0.05). The 2^nd^ and 3^rd^ youngest full-sized leaves of four plants were sampled and combined per treatment and per timepoint. The relative expression in Adj at 0 dpi was set as 1 and the others were adjusted accordingly. The error bars represent the standard error of the mean. **C.** Diphenylene iodonium (DPI) partly abolishes the PS3-IR to *P. viticola* in grapevine. Leaf disks were treated during 1 day with 10 µM DPI, washed and then treated with 2.5 g l^−1^ PS3 during 1 day, washed and, finally inoculated with *P. viticola*. Leaf sporulating area evaluated at 8 dpi. Different letters indicate statistically significant differences (*P*<0.05; ANOVA followed by LSD test). Data are representative of three independent experiments (n = 3). **D.** Microscopic analyses on the same grapevine leaf discs show that DPI inhibits the primed H_2_O_2_ production (black arrows and inset) and callose deposition (white arrows and inset) during PS3-IR, leading to *P. viticola* spreading. Aniline blue and DAB staining were realized to detect callose and H_2_O_2,_ respectively. Pictures are representative of three independent experiments. Bar = 100 µm.

PS3-IR is correlated with the priming of defense responses after *P. viticola* inoculation, including the SA-dependent pathway (Figure 3) and with a specific H_2_O_2_ production, callose deposition and HR-like cell death [30] (Figure S3). Therefore, gene expression of a well-established ROS-related gene, *Respiratory burst oxidative homolog D (RbohD*) [21] and a HR marker gene, *HSR203J* [43] were analyzed by qPCR in PS3-treated plants during downy mildew infection. At 1 dpi, PS3-treatment triggered a significantly higher gene expression of *RbohD* and *HSR203J* in response to *P. viticola* (Figures 4A, B) that correlated with H_2_O_2_ production and HR-like cell death detected at the microscopic level (Figure S3). The NADPH oxidase inhibitor diphenylene iodonium (DPI) was applied to understand the significance of the observed ROS priming, in PS3-treated and *P. viticola* inoculated plants. Pre-incubation with DPI did not alter the pathogen infection process (Figure 4C). Co-treatment with DPI and PS3 significantly reduced the PS3-IR in grapevine (Figure 4C). At the microscopic level, callose deposition and HR-like cell death were not observed anymore in PS3+ DPI co-treated and inoculated leaves (Figure 4D).

### Plasma Membrane Depolarization and ROS Priming are Key Components of the PS3-induced Resistance

As PS3 triggered a plasma membrane depolarization (Figure 1D), we hypothesized an existing link between ion channel activity, plasma membrane depolarization, H_2_O_2_ production and HR-like cell death during the PS3-IR against *P. viticola*.

**Figure 5:**
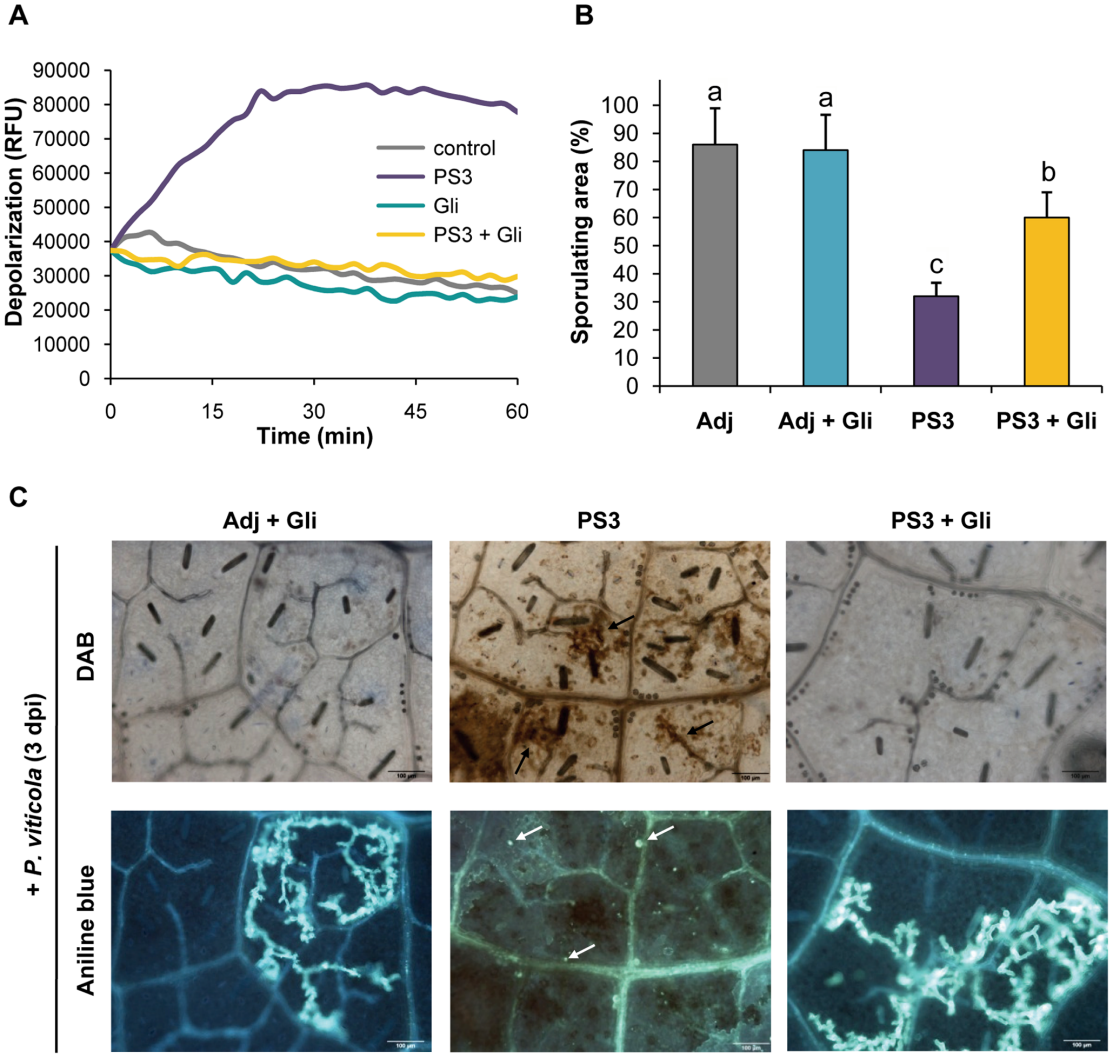
Plasma membrane depolarization mediates the primed ROS production during the PS3-IR to *P. viticola*. **A.** The anionic channels inhibitor glibenclamide (Gli, 200 µM) blocked the plasma membrane depolarization triggered by PS3 in grapevine cell suspensions revealed by the DIBAC_4_ probe fluorescence. **B.** Sporulating areas indicate that Gli blocked the PS3-IR to *P. viticola* in grapevine leaf discs. Leaf discs were treated during 24 h with Gli (200 µM), washed and then treated with 2.5 g l^−1^ PS3 during 24 h, washed and, finally inoculated with *P. viticola.* Leaf sporulating area evaluated at 8 dpi. Different letters indicate statistically significant differences (*P*<0.05; ANOVA followed by LSD test). Data are representative of three independent experiments (n = 3). **C.** Microscopic analyses on the same grapevine leaf discs show that Gli inhibits the primed H_2_O_2_ production (black arrows) and callose deposition (white arrows) during PS3-IR, leading to *P. viticola* spreading. Aniline blue and DAB staining were realized to detect callose and H_2_O_2,_ respectively. Pictures are representative of three independent experiments. Bar = 100 µm.

To assess the involvement of anionic channel activity in the PS3-induced plasma membrane depolarization, the glibenclamide anion channel blocker (Gli) was used [6], [7]. Compared to control cells, Gli inhibitor suppressed the depolarization induced by PS3 to near control levels (Figure 5A). These data suggest that PS3 elicited a rapid and sustained plasma membrane depolarization through activation of anion channels. Similar results were also obtained with niflumic acid (Nif), another structurally unrelated anion channel inhibitor (data not shown).

To evaluate whether the activation of anionic channels is a determining step in the regulation of PS3-IR, the effect of Gli inhibitor was examined by observing the *P. viticola* development in PS3-treated plants. Figure 5B shows that pre-incubation with Gli did not alter the pathogen infection process whereas PS3 induced a strong reduction of the sporulating surface. Co-treatment with Gli and PS3 significantly reduced the PS3-IR (Figure 5B).

To confirm these results, microscopy was used to reveal H_2_O_2_ formation, callose deposition and *P. viticola* development in each modality. Interestingly, DAB and aniline blue staining indicate that co-treatment PS3+ Gli abolished the priming of H_2_O_2_ production and callose deposition leading to highly infected grapevine tissues (Figure 5C). These results suggest that the primed ROS production depends on an upstream channel activity and regulates downstream callose deposition and HR-like cell death, key components of the PS3-IR.

## Discussion

### Sulfation of Laminarin Improves the β-glucan Activity

In plants and mammals, the fact that oligosaccharides must carry crucial sulfates for their biological function suggests that chemical sulfation of oligosaccharides can improve their biological properties. Compared to Lam, its sulfated derivative PS3 triggered an enhanced immunity against *P. viticola* in *V. vinifera* and a stronger immunity against TMV in *Nicotiana tabacum* [29]. As for drug discovery, our results indicate that the chemical modification of an elicitor, such as PS3, could improve its resistance-inducer efficiency. In a structure–activity analysis, Ménard *et al.* [29] demonstrated that the sulfate residues and a minimum β-1,3 glucan chain length (DP>5) were essential for PS3 activity in tobacco. Moreover, if Lam is a substrate for plant β-1,3 glucanase, its sulfation clearly protects the molecule from its enzymatic degradation [44]. In tobacco plants, Klarzynski *et al*. [28] have shown that while Lam elicits phenylalanine ammonia lyase (PAL) activity, di-, tri- and tetramers of β-1,3 glucan were inactive. Thus, a basal activity of plant glucanases can degrade Lam and consequently releases short inactive β-glucans whereas PS3 still remains an active molecule during a longer period. This might explain the higher resistance induced by PS3 compared to Lam.

### PS3 Directly Triggers a Sustainable Plasma Membrane Depolarization

Studies of early signaling events indicated that, in contrast to Lam, PS3 did not directly trigger typical elicitor-induced signaling events such as cytosolic [Ca^2+^] variations, oxidative burst and activation of 2 MAPKs. These data clearly indicate that sulfation of Lam modifies some defense-related events in grapevine as it has been previously observed in tobacco and Arabidopsis [29]. It is not surprising that PS3 was unable to elicit these three signaling events as Ca^2+^ is known to act upstream of MAPK activation and ROS production [4]. Interestingly, BABA, another priming agent, is also unable to elicit cytosolic [Ca^2+^] variations, ROS production or MAPK activation [21]. Thus, screenings to identify powerful resistance inducers should not be realized only on early signaling events. Supporting this conclusion, PS3 and BABA were shown to trigger a strong grapevine induced resistance against downy mildew without eliciting any ROS production or MAPK activation (this study and [21]). The fact that cytosolic [Ca^2+^] variations are induced by Lam but not by PS3 suggests that ion fluxes trough plasma membrane are modified depending on the β-glucan structure. In this study, it has been shown for the first time that the priming agent PS3 directly triggers a sustained plasma membrane depolarization while Lam only triggers a transient one. In soybean roots, β-glucans also trigger a plasma membrane depolarization varying with β-glucan structure: short DP 5 has no effect, DP 7–15 induces a transient depolarization whereas DP>15 trigger the strongest depolarization [45]. Knowing that Gli and Nif inhibit the PS3-triggered plasma membrane depolarization indicates that anionic channel activity is involved in PS3 signaling in grapevine. In tobacco, the *Phytophthora* elicitor cryptogein also triggers a sustained plasma membrane depolarization that can be blocked by Gli or Nif anionic channel inhibitors [7]. A pharmacological approach has shown that this anion efflux was important signaling event acting upstream of induction of defense-related genes (*i.e. PAL, HSR203J*) and HR-like cell death [6].

### PS3 Directly Induces a Stress-responsive Transcriptome

Our study also indicates that PS3 directly up-regulates 132 genes in grapevine. Interestingly, microarray analysis shows that 94% of the genes induced by Lam (88/94) are also up-regulated by PS3. However, the grapevine transcriptome was more affected by PS3 treatment than by Lam as PS3-treated plants exhibit more induced genes (132 *vs* 94) and a higher global fold change. For example PS3 highly induces the expression of a putative *SAMT1* which might result in the production of methylsalicylate (MeSA), a mobile plant immune signal [46]. The different regulation of the JA/SA balance observed after PS3 or Lam treatment might be due to the higher induction of specific transcription factors such as WRKY40 [47]. So, compared to Lam, PS3 induces a stronger plasma membrane depolarization correlated to stronger transcriptome activation and higher induced resistance.

Moreover, transcriptome analysis provided evidence that PS3 modulates grapevine immunity through induction of a stress-responsive transcriptome.

Many of these genes encode chloroplast-targeted proteins corroborating many earlier results indicating that light and photosynthetic metabolites are necessary for development of resistance, notably by stimulating the oxidative burst and the HR [48]. The GO enrichment analysis also showed that PS3 induces genes involved in glycolysis to provide energetic metabolites. Among them, glycerol-3-phosphate (G3P) has been discovered to be a key mobile signal during induced resistance in Arabidopsis [49]. Altogether, these data indicate that the PS3-transcriptome might provide energetic metabolites and mobile immune signals to anticipate pathogen attack *via* faster synthesis of stress-responsive transcripts. However, if different grapevine resistance inducers (PS3; thiamine and the beneficial microorganism *Trichoderma harzianum* T39) up-regulate common stress/defence-related genes after downy mildew infection [30], [50], [51], it seems that the mode of action could be different. Indeed, the genes directly modulated by T39 or PS3 are different. In particular, T39 up-regulates genes involved in signal transduction process and ET-related genes [50] whereas PS3 rather triggers HR-related and SA-modulated genes (Figure 2). If PS3 and thiamine both induced the expression of genes of the phenylpropanoid pathway, PS3 induced the accumulation of phenolic and phytoalexin metabolites only after downy mildew inoculation whereas thiamine directly elicited this production after treatment [30], [33], [51]. However, after *P. viticola* infection, similar defence genes are primed by the different resistance inducers, including *Chitinase 1b*, *PR-2*, *LOX-9*, *GST*, *PAL*, *STS* and *ROMT* [30], [33], [50], [51]. Recently, Tsai *et al*. [35] have also shown that BABA directly up-regulates a stress-responsive transcriptome in Arabidopsis (20% compared to 5% in the whole genome). Interestingly, many *HSP/HSC* genes are up-regulated by PS3 in *V. vinifera* and by BABA in Arabidopsis [35]. These chaperone proteins have been reported to play a key role in plant immunity by promoting the stability of disease resistance proteins [52], [53] and by priming gene transcription and systemic acquired resistance [54]. It has also been reported that the HSP90 chaperone machinery mediates posttranscriptional gene silencing (PTGS) *via* formation of RNA-induced silencing complexes (RISCs) that contain ARGONAUTE (AGO) [55]. The fact that *HSP90/HSC70* and *AGO1* are more induced by PS3 than by Lam suggests a more efficient PTGS triggered by PS3. This difference could explain that Lam directly elicited immune responses whereas PS3 only primed them. Moreover, it has been recently shown that *B. cinerea* small RNAs suppress plant immunity by hijacking host RNA interference machinery *via* their binding to plant AGO1 [56]. As *P. viticola* might also produce small RNAs effectors, future works will be necessary to understand if AGO1 is important for the priming phenomenon and for resistance against this eukaryotic pathogen.

### PS3 Primes SA-dependent Defense Pathway Upon Downy Mildew Inoculation

In tobacco, PS3-IR against TMV has been shown to be correlated to the elicitation of the SA-dependent defense pathway [29]. Upon *P. viticola* inoculation, PS3 primes a significant SA accumulation and the expression of the 2 SA-marker genes *NRX1* and *PR-2*. In tobacco and Arabidopsis, PS3 also induced SA accumulation and expression of SA-dependent PR proteins [29]. Globally, these results suggest that PS3-IR commonly involves the SA signaling pathway, although PS3 showed elicitor or priming effects depending on the plant species. Similarly, BABA directly elicits plant defenses in tomato [57] while it primes them in Arabidopsis [22]. The fact that different studies show that the SA-analogue BTH is able to induce resistance against *P. viticola* in grapevine (this study; [58], [59]) clearly indicates that the SA-pathway efficiently contributes to trigger resistance against this biotrophic oomycete. Previous results showing that the expression of the *9-lipoxygenase (9-LOX)* gene is primed during PS3-IR [30] and that the LOX inhibitor 5, 8, 11, 14-eicosatetraynoic acid led to a reduced IR against *P. viticola* after BABA or PS3-pretreatment [30], [60] suggest the coordinated involvement of the oxylipin-pathway. If the involvement of the JA-dependent pathway has been suggested for grapevine resistance against downy mildew [60], [61], its quantification by LC-MS and the expression of JA-marker genes (*LOX-A, JAZ1*) were not statistically significant to definitely conclude about its role in PS3-IR. The fact that SA suppresses the JA signaling pathway in Arabidopsis [62] suggests that a similar prioritization might also exist in grapevine during PS3-IR. Further experiments will be needed to investigate the finely tuned relationship between these two hormones in *V. vinifera*.

### Priming of H_2_O_2_ Production, Callose Deposition and HR-like Cell Death during PS3-IR are Dependent on Plasma Membrane Depolarization

Our work has demonstrated that the PS3-IR in grapevine to *P. viticola* was correlated to the priming of defense genes, H_2_O_2_ production and HR-like cell death (this study; [30]). Following infection, H_2_O_2_ accumulation is one of the defense responses commonly observed in primed plants [21], [63]–[65] and have been demonstrated to be involved in the callose deposition and HR cell death in different studies [66]–[69]. Moreover, the callose deposition has been commonly reported to play a key role in the grapevine induced resistance against *P. viticola* [30], [69], [70]. Treatment with DPI, a NADPH oxidase inhibitor, increased significantly the susceptibility of PS3-primed plants to downy mildew indicating that inhibition of the primed H_2_O_2_ accumulation affected the PS3-IR. Nevertheless, it cannot be excluded that H_2_O_2_ might be produced by other sources of ROS. The capacity of a resistance inducer to directly elicit the H_2_O_2_ production doesn’t seem to be crucial for the IR compared to the priming of H_2_O_2_ production observed in response to *P. viticola* inoculation in treated plants (this work, [70]).

Plasma membrane depolarization has been observed to be an initial feature of apoptosis in mammals and HR-like cell death in plants [7]. Since anionic channels play key roles in plasma membrane depolarization, Gli was used to determine the contribution of channel activity during the plasma membrane depolarization preceding the PS3-IR to downy mildew. In the tobacco-cryptogein model, inhibition of the NO_3_^−^ efflux blocked the H_2_O_2_ production and reduced HR-like cell death [6]. In grapevine, treatment with Gli also inhibited the priming of H_2_O_2_ production, callose deposition and reduced the PS3-IR showing that channel activity acts upstream of defense reactions involved in the PS3-IR.

On the whole this study reports that plasma membrane depolarization plays an initial signal transduction role leading to primed SA-dependent defenses, ROS production and HR-like cell death. Previously a pharmacological approach has also shown that lipoxygenase activity and callose deposition were involved in PS3-IR [30]. H_2_O_2_ production, callose deposition and stilbene production have also been found to be associated to the resistance against *P. viticola* triggered by other inducers [51], [69]. Altogether, our results indicate that PS3-IR against *P. viticola* needs anionic channels and lipoxygenase activities to prime ROS and SA production, callose deposition and HR like cell death. This study has also provided β-glucan-, SA- and JA- marker genes in grapevine and new genes putatively involved in the priming phenomenon that should be further characterized by a functional genomics strategy.

In term of vineyards application, one should keep in mind that our results were obtained on the 2^nd^ and 3^rd^ fully expanded leaves from greenhouse-grown plants that are the most responsive to PS3-IR [33]. Younger or older leaves are less PS3-responsive and thus global protection in the vineyard should not be extrapolated for the moment.

## Supporting Information

Figure S1 **β-1,3 glucans induce resistance in grapevine against** ***Plasmopara viticola*** **.**
(PDF)

Figure S2 **Quantification of JA and SA in uninfected grapevine plants treated with PS3, Lam, or adjuvant.**
(PDF)

Figure S3 **Primed-H_2_O_2_ and callose deposition observed during PS3-IR.**
(PDF)

Table S1 **List of grapevine modulated genes in response to PS3, Lam, SA and JA at 12 hpt.** Large data set in xls file can be downloaded.
(XLSX)

Table S2 **Specific and common genes modulated 12 hpt with PS3, Lam, SA and JA described in the Venn diagram.** Large data set in xls file can be downloaded.
(XLSX)

Table S3 **Gene list corresponding to the GO enrichment analysis by the AgriGO software.** Large data set in xls file can be downloaded.
(XLSX)

Table S4 **Sequences of primers used for qPCR experiments.**
(PDF)
