# Correction: The Sulfated Laminarin Triggers a Stress Transcriptome before Priming the SA- and ROS-Dependent Defenses during Grapevine's Induced Resistance against *Plasmopara viticola*

**DOI:** 10.1371/journal.pone.0194327

**Published:** 2018-03-08

**Authors:** Adrien Gauthier, Sophie Trouvelot, Jani Kelloniemi, Patrick Frettinger, David Wendehenne, Xavier Daire, Jean-Marie Joubert, Alberto Ferrarini, Massimo Delledonne, Victor Flors, Benoit Poinssot

The Adj + Gli panel in Fig 5C incorrectly appears as a duplicate of the Adj + DPI panel in Figure 4D. This error arose during the construction of Fig 5. Please see the corrected Fig 5 here.

**Fig 5:**
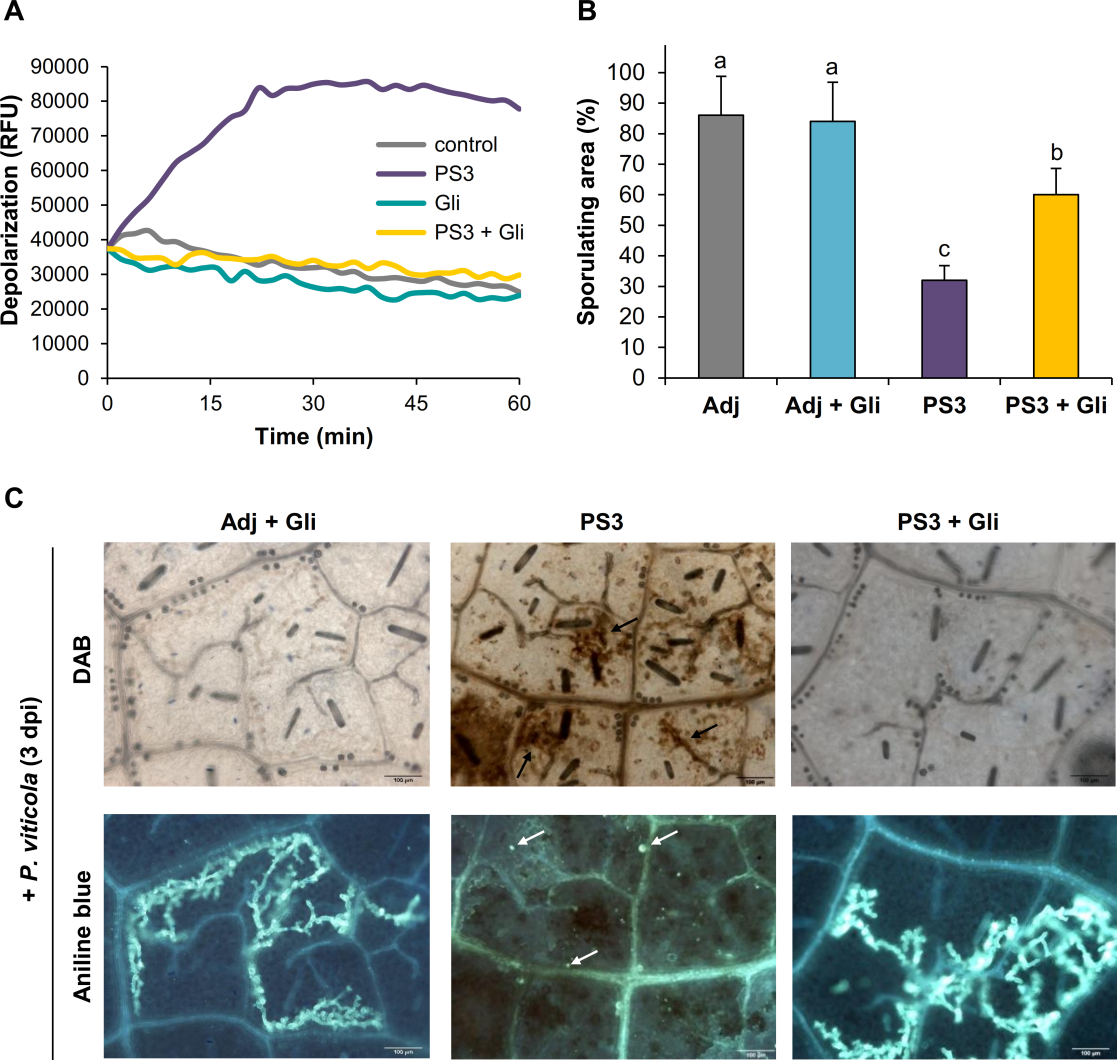
Plasma membrane depolarization mediates the primed ROS production during the PS3-IR to *P*. *viticola*. **A.** The anionic channels inhibitor glibenclamide (Gli, 200 μM) blocked the plasma membrane depolarization triggered by PS3 in grapevine cell suspensions revealed by the DIBAC_4_ probe fluorescence. **B.** Sporulating areas indicate that Gli blocked the PS3-IR to *P*. *viticola* in grapevine leaf discs. Leaf discs were treated during 24 h with Gli (200 μM), washed and then treated with 2.5 g l^−1^ PS3 during 24 h, washed and, finally inoculated with *P*. *viticola*. Leaf sporulating area evaluated at 8 dpi. Different letters indicate statistically significant differences (*P*<0.05; ANOVA followed by LSD test). Data are representative of three independent experiments (n = 3). **C.** Microscopic analyses on the same grapevine leaf discs show that Gli inhibits the primed H_2_O_2_ production (black arrows) and callose deposition (white arrows) during PS3-IR, leading to *P*. *viticola* spreading. Aniline blue and DAB staining were realized to detect callose and H_2_O_2_, respectively. Pictures are representative of three independent experiments. Bar = 100 μm.
