# Supplementary material for: The Sulfated Laminarin Triggers a Stress Transcriptome before Priming the SA- and ROS-Dependent Defenses during Grapevine's Induced Resistance against Plasmopara viticola
Source: PLoS One. 2014 Feb 6;9(2):e88145. doi: 10.1371/journal.pone.0088145 (PMC3916396; doi:10.1371/journal.pone.0088145)
Supplement: Figure S1 — β-1,3 glucans induce resistance in grapevine against Plasmopara viticola . (PDF) [file pone.0088145.s001.pdf]

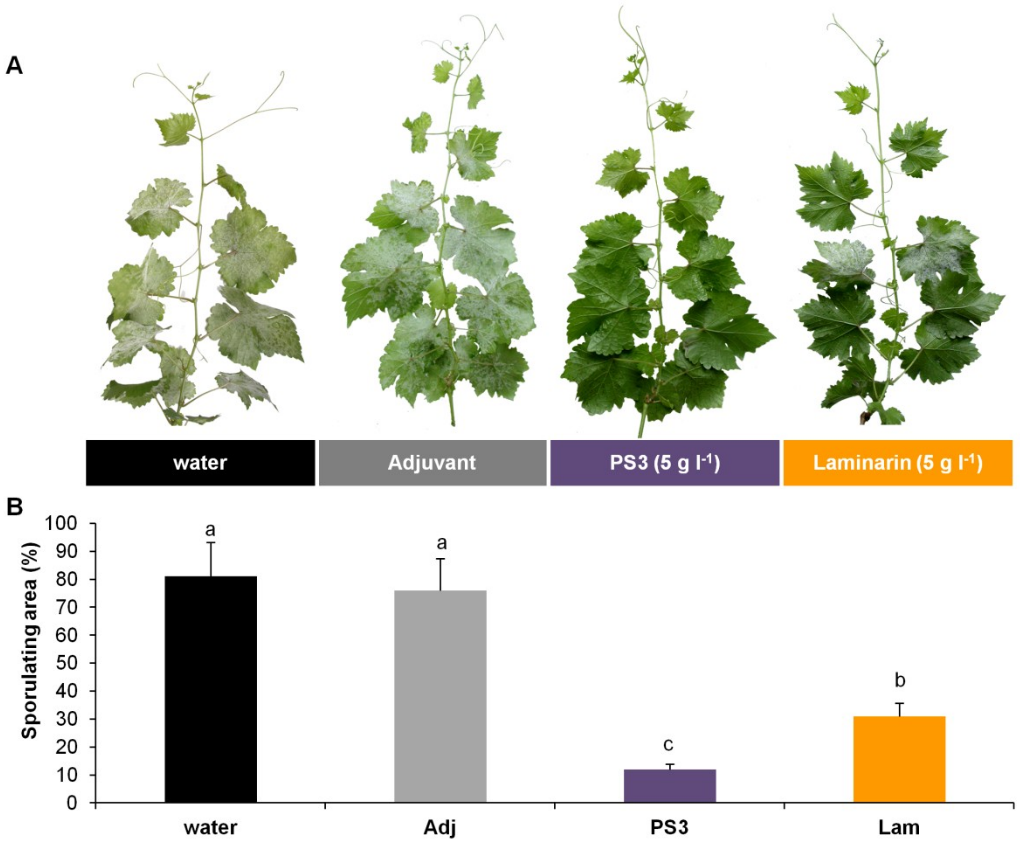

Figure S1. β-1,3 glucans induce resistance in grapevine against *Plasmopara viticola*. Plants were sprayed with water, adjuvant (0.05%), PS3 (5 g l^{-1}) or Lam (5 g l^{-1}) before *P. viticola* inoculation 2 days later (10^4 spores ml^{-1}). **A.** Visible disease symptoms at 8 dpi on lower face of the leaves. **B.** Leaf sporulating area evaluated at 8 dpi (%). Data represent means ± SD from 6 plants per modality (n=6). Different letters indicate statistically significant differences (*P*<0.05; ANOVA followed by LSD test). Results are from one representative experiment out of 5 repetitions with similar results.
