# Supplementary material for: The Sulfated Laminarin Triggers a Stress Transcriptome before Priming the SA- and ROS-Dependent Defenses during Grapevine's Induced Resistance against Plasmopara viticola
Source: PLoS One. 2014 Feb 6;9(2):e88145. doi: 10.1371/journal.pone.0088145 (PMC3916396; doi:10.1371/journal.pone.0088145)
Supplement: Figure S2 — Quantification of JA and SA in uninfected grapevine plants treated with PS3, Lam, or adjuvant. (PDF) [file pone.0088145.s002.pdf]

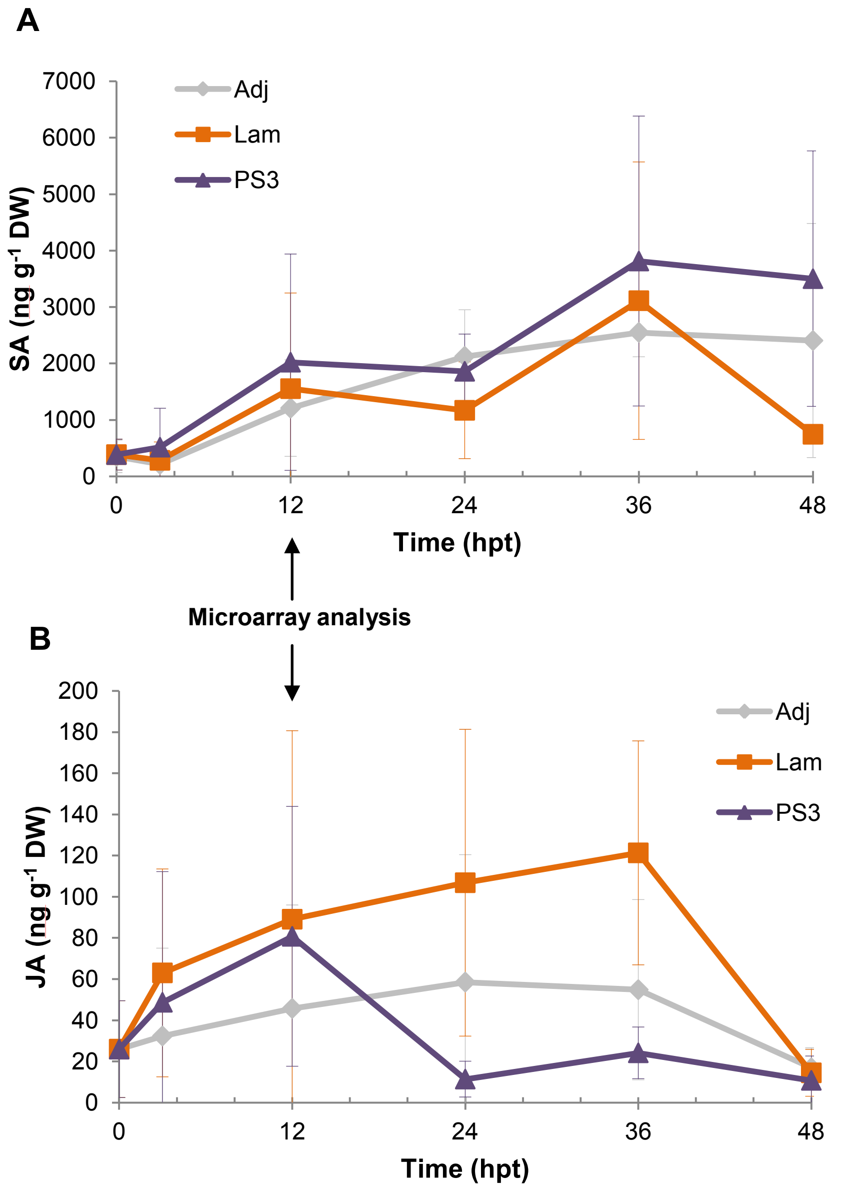

Figure S2: Quantification of SA and JA in uninfected grapevine plants treated with PS3, Lam, or adjuvant.

Endogenous SA and JA were quantified by LC-MS in grapevine plants during 48 hours post-treatment (hpt) with PS3, Lam or adjuvant (Adj). No statistical difference (P<0.05, student t test) has been found comparing PS3 *vs* Adj or Lam *vs* Adj from three biological independent experiments (n=3).
