# Supplementary material for: The Sulfated Laminarin Triggers a Stress Transcriptome before Priming the SA- and ROS-Dependent Defenses during Grapevine's Induced Resistance against Plasmopara viticola
Source: PLoS One. 2014 Feb 6;9(2):e88145. doi: 10.1371/journal.pone.0088145 (PMC3916396; doi:10.1371/journal.pone.0088145)
Supplement: Figure S3 — Primed-H2O2 and callose deposition observed during PS3-IR. (PDF) [file pone.0088145.s003.pdf]

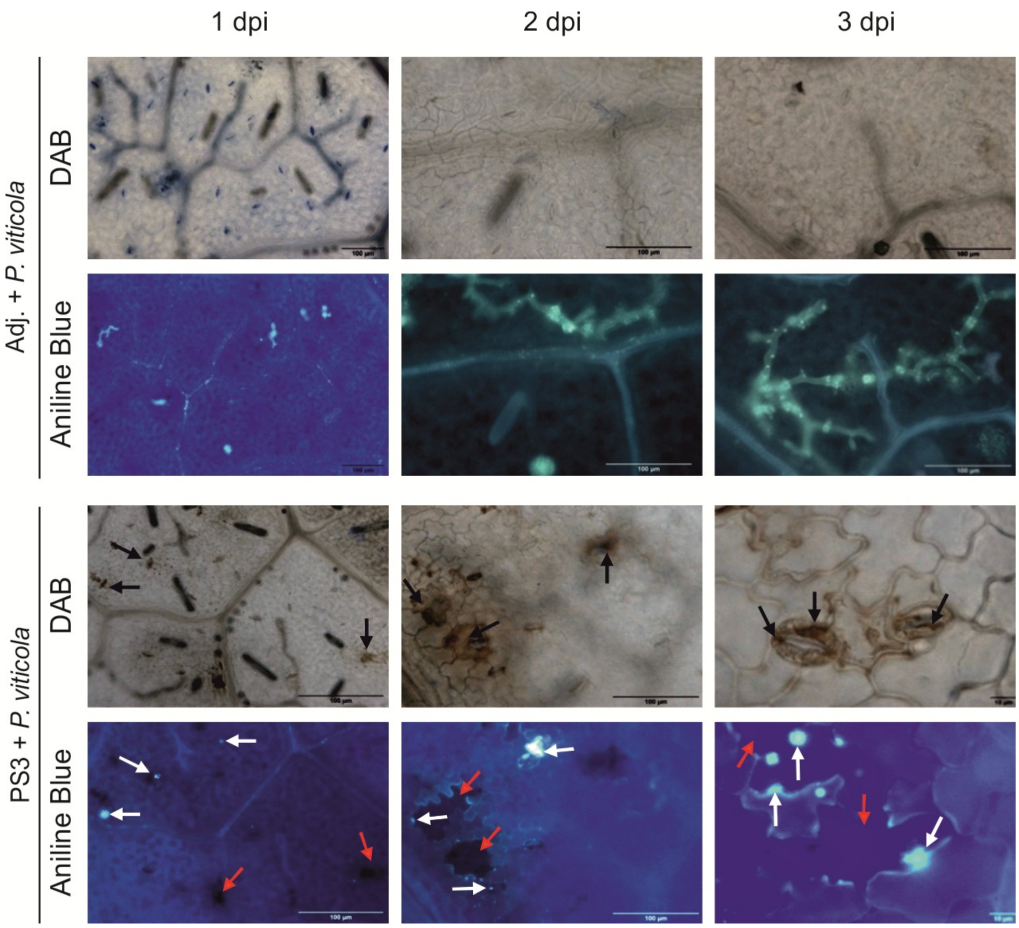

Figure S3. Primed-H_2O_2 and callose deposition observed during PS3-IR. Primed H_2O_2 production and callose deposition are specifically observed during PS3-IR at 1, 2 and 3 dpi with *P. viticola*, as revealed by 3,3'-diaminobenzidine (DAB) or aniline blue staining, respectively. Pictures are representative of three independent experiments. Black arrows indicate brown precipitates due to H_2O_2 production and DAB staining. White arrows indicate callose deposition revealed by aniline blue staining. Red arrows indicate dead cells. Bar = 100 or 10 μm.
