# Supplementary material for: The Sulfated Laminarin Triggers a Stress Transcriptome before Priming the SA- and ROS-Dependent Defenses during Grapevine's Induced Resistance against Plasmopara viticola
Source: PLoS One. 2014 Feb 6;9(2):e88145. doi: 10.1371/journal.pone.0088145 (PMC3916396; doi:10.1371/journal.pone.0088145)
Supplement: Table S4 — Sequences of primers used for qPCR experiments. (PDF) [file pone.0088145.s007.pdf]

Table S4: **Sequences of primers used for qPCR experiments.**

Gene	Forward primer	Reverse primer	NCBI identifier
PR-2	TCAGCCGTCCTCGGCAAATCA	TTGGCCAGGAGTGGGGAGCC	XM_002278087
NRX-1	CTGAGAAGGTGAAGCATGCT	CTCTCAAACTCATTGTGCTATT	XM_002262821
RbohD	ACCATGCTTCAGTCCCTCCAT	AGCGATCTTCTTGAAGACTTGTCGCC	XM_002268568
HSR203J	TGGAGGAAACATCGTTCACA	CCTGGACAATTCTGCCATCT	XM_002285050
VATP16	CTTCTCCTGTATGGGAGCTG	CCATAACAACTGGTACAATCGAC	XM_002269086
